# Parental digital health information seeking behavior in Switzerland: a cross-sectional study

**DOI:** 10.1186/s12889-019-6524-8

**Published:** 2019-02-21

**Authors:** Rebecca Jaks, Isabel Baumann, Sibylle Juvalta, Julia Dratva

**Affiliations:** 1 Departement Gesundheit, ZHAW Zürcher Hochschule für Angewandte Wissenschaften, Institut für Gesundheitswissenschaften, Technikumstrasse 71 Postfach, CH-8401 Winterthur, Switzerland; 2 National Center of Competence in Research “Overcoming Vulnerability: Life Course Perspectives”, Winterthur, Switzerland; 3 Medical Faculty, University of Basel, Basel, Switzerland

**Keywords:** Digital media, Child health, Parenting, Information seeking, Health information sources, Internet

## Abstract

**Background:**

Digital media are increasingly abundant and used to seek health information, however, to date very little is known on parents’ seeking behavior in the context of child’s health and development outside English-speaking and Scandinavian countries. By investigating the prevalence of, and reasons for use, we studied parents’ perception of the Internet as a resource for improving their health-related knowledge.

**Methods:**

The survey was conducted in a random sample of 2573 Swiss-German parents with at least one child aged less-than 2 years old. Parents received a mailed invitation to fill in an online questionnaire. Two reminders were sent, the later with a paper questionnaire attached. The questionnaire included questions on use of print, digital, and personal information sources, as well as different information situations: general health and development or illness. We ran descriptive analyses on information seeking behavior, type of digital media used, reasons of use. We also conducted regression analyses to explore factors associated with parental perceptions with regard to the Internet’s utility as a source for health information.

**Results:**

A total of 769 questionnaires were returned (response rate 30%). Nearly all parents (91%) used digital media for seeking information on their child’s health and development, and the main reason for use was indicated as being the 24/7 availability of information. Search engines (55%) and webpages for parents (47%) were by far the most frequently used digital media. Generally, the internet is perceived as a good resource, especially by fathers (OR = 1.80, 95% CI: 1.03–3.16). However, a large percentage of parents are skeptical about the correctness of online info (91%), are unsure about their interpretive understanding, and ask for guidance from their pediatrician (67%).

**Conclusions:**

The Internet has become a highly frequented source of information for Swiss-German parents on children’s health with largely valuable perceptions of its utility. Digital media are used in addition to and not in replacement of print media and personal contacts. Increasing parental guidance by health and public health professionals could improve parental digital health utilization and empower parents in the new role they adopt.

**Electronic supplementary material:**

The online version of this article (10.1186/s12889-019-6524-8) contains supplementary material, which is available to authorized users.

## Background

The transition to parenthood is undoubtedly a major life event that has important implications for parents. The birth of a child is associated with profound changes and can therefore frequently be very stressful for mothers and fathers [1–3]. Parents often feel inadequately prepared for this new situation [4–6].

Recent studies have shown that the Internet, and digital media in general, are used more and more by expecting and new parents as a source for health information [7–10]. The current generation of young parents predominantly grew up with digital media. It is therefore not surprising that they increasingly search online to obtain information about their child’s health and development. This trend is reflected in the increased number of websites designed for parents [11], offering access to a wide range of topics on children, health, and parenthood and in some cases social exchange [10].

The advantages of accessing health information on the Internet are manifold. The web makes it possible to overcome spatial and temporal barriers by allowing individuals to obtain information 24/7. This availability has been indicated as-being particularly appreciated by mothers, as they often feel the need to have information to hand immediately, especially when it concerns the health of their baby [12]. Other positive aspects are the possibility to exchange experiences and express opinions in an anonymous setting without feeling judged, and the opportunity to find social support from other parents via virtual communities [12, 13].

Despite the great potential of the Internet, the quality of the information available online is questionable. Literature indicates that online health information differs considerably in reliability and that incorrect or inaccurate information is common [14–16]. Often parents do not fully trust the information accessed online [10]. In fact, a study by Kind et al. [17] yields that parents prefer to discuss the information found on the web with their physician.

Previous research on digital media use has focused predominantly on English-speaking and Nordic countries, and, to our knowledge, parents’ use of digital media to obtain information on child’s health and development has not yet been investigated in Switzerland [18, 19]. Our survey examined the digital media behavior and reasons of use in the context of child’s health and development of Swiss parents, living in the German-speaking part of the country, who had children aged 0 to 2 years. Further, we investigated if parents perceive the Internet as a good resource for improving their health knowledge.

## Methods

### Study population

Our study population consists of a population-based sample of parents with a child aged <− 2 years. Names and addresses of the 2573 mothers with children born in the preceding 24 months were selected randomly by birth registries of the City Zürich and through convenience sample from small municipalities in the same region municipalities in order to include both urban and rural communities in the German-speaking part of Switzerland (75%/25%). Given that the study is the first to investigate information seeking behavior in the context of child health and development in Switzerland, the sample size (*N* = 2500) was calculated to ensure a prevalence estimation of digital media use with an α = 0.05 and a precision of ±2.5% based on a conservative assumption of a 50% prevalence [20]. The ethical commission of the Canton of Zurich confirmed an exemption from the ethics review (BASEC Req-2017-00817).

The study includes a quantitative questionnaire, data of which are presented in this paper, and a qualitative part consisting in a focus group with parents and interviews with pediatricians. In the current paper only quantitative results are presented. The data was collected between January and May 2018. We sent an invitation letter with a link to the online questionnaire to parents. To increase the response rate, together with the second and last reminder letter, we sent a paper version of the questionnaire. Parents were informed on the aims of the study, their free choice in participation and that data was collected anonymously without any identification code. They consented to participating by returning the filled-in questionnaire.

### Questionnaire

The questionnaire consisted of seven parts: (1) Socio-demographic information of survey participant, (2) and child, (3) use of print or digital media and personal sources with regard to child’s health and development, (4) and child’s acute health problems, (5) information behavior around last pediatric visit, (6) e-health literacy of survey participant, attitude towards online health information and use for personal health-related information, and (7) health status of survey participant (see Additional file 1 for full questionnaire). To ensure overall understanding, completeness of answer items, and the functioning of technical aspects the online questionnaire was pretested by parents, eligible for study participation. Their data is not included in the final analysis.

Child health related questions were based on the German Health Interview and Examination Survey for Children and Adolescents [21]. Parental education was measured using a question from the Swiss Infant Feeding Study [22], collapsing the categories ‘no education’, ‘only compulsory education’, and ‘secondary education’ to one single category ‘lower education’ for reasons of frequency in the population sample. Questions on attitudes towards online health information and frequency of online search were based on the Flash Eurobarometer 404 on European citizens’ digital health literacy [23], and those measuring trust, assessing correctness, and understanding were taken from the study by Wainstein et al. [9].

Parents were asked about their use of the following information sources: 1. digital media, such as social media, webpages for parents, apps, search engines, webpages of pediatricians or children’s hospitals, and official webpages of health services or health organizations; 2. print media, such as books, magazines, newspapers, and other print media; and 3. formal and informal personal contacts, namely pediatrician, other health professionals, telephone consultation of a children’s emergency service or hospital, telephone consultation of the health insurance, family members, or friends, neighbor, and other acquaintances. Each item provided five response options: ‘never’, ‘rarely’, ‘sometimes’, ‘frequently’, and ‘very frequently’. A binary variable for frequency of use was constructed for each information source listed: taking the value 1 if the use was ‘frequent’ or ‘very frequent’ and 0 otherwise.

To calculate general print media and personal contacts use, we defined participants as “non-users” if a parent answered “never” to all items of the respective information source and otherwise as “users”. For general digital media use, the questionnaire included a filter question about digital media use relating to child health issues. Parents responding ‘yes’ were defined as digital media users. For digital media use with regard to child’s health and development, a score defining frequency of use and another for multimodality were created in addition to the binary frequency variable. We summarized the response option chosen by the parent for each of the six different digital media, attributing 0 to ‘never’ up to 1 for ‘very frequently’. For the multimodality score the number of digital media used, irrespective of the frequency, was calculated, ranging from one to six.

The use of social media, apps, websites and, chats, posts, or forums was investigated in more detail in digital-users. Parents reported the frequency of use, ranging from 0 ‘never’ to 4 ‘very frequently’, of each social media (Facebook, Instagram, Twitter, YouTube, Pinterest, and professional social networks [LinkedIn]). Parents were asked to tick off a maximum of three apps they used most frequently from the list of websites provided in the survey. Lastly, they could indicate if they were consumers and/or contributors of information in chats, of posts, or forums.

To assess parents’ perception about the Internet as a good resource we asked all parents to indicate how much they agree with the statement “*the Internet is a good tool to help improve my knowledge of health-related topics*”. Answers ranged from ‘completely agree’ to ‘completely disagree’ on a four point scale and included the option ‘I don’t know’. A binary variable “good resource” was created, taking the value 1 if the respondent agreed with the statement and 0 if she or he disagreed. Parents answering ‘I don’t know’ were excluded from this analysis. Additional questions addressed trust, correctness, and understanding of information obtained from the Internet as well as parent’s wish for digital guidance by their pediatrician.

A binary variable for child’s disability was created. The child was considered disabled if parents reported one of the following health problems: physical impairment of health (e.g. malformation), developmental delay, hearing or visual impairment, or congenital disability.

Factors included in the explorative ordered logistic regression were chosen based on existing literature and underlying hypotheses.

### Statistical analyses

Univariate descriptive statistics were applied to describe the sociodemographic characteristics of the study population and their youngest child as well as the use of the different health information sources.

We performed bivariate analyses to evaluate differences in frequency of digital media use by parental characteristics: sex, age, parental education and first child (chi-square tests and one-way ANOVA, 95% confidence interval). The variable age proved non-significant in group analyses and had due to a high percentage of missing data (18%) we did not include the variable in the multivariate analyses.

Explorative ordered logistic regression models were run to investigate associations between the outcome variable “good resource” and the exposure variables parental education, sex, first child, age of child, disability, parental Internet use for health-related topics during the last 12 months, and frequency score of digital media use. Accordingly, results were expressed as odds ratios with a 95% confidence interval (ORs, 95% CI). Statistical analyses were performed using Stata Version 15.1 [24]. To control for potential bias due to exclusion of non-users of digital media we calculated a sensitivity analysis with the variable general digital media use (yes/no) instead of the digital frequency score.

## Results

Overall, 842 individuals responded to the survey of which 429 (56%) responded to the online and 340 (44%) to the paper questionnaire. Seventy-three responses had to be deleted in the data cleaning process; leading to a total of 769 data sets, which represent a response rate of 30%. Reasons for exclusion were: empty questionnaire (*N* = 31), missing answers to key questions (*N* = 40), one non-plausibility of key questions, and one double entry.

### Study sample characteristics

The majority (88%) of the respondents were mothers, the mean parental age was 35.7 years (SD = 4.3), and 71% were of Swiss nationality (Table 1). Two-thirds (76%) fell in the category ‘higher education’ and 42% indicated a monthly net household income of over 9000 Swiss francs (CHF). For slightly more than half of the respondents (52%) it was their first child. 49% of the infants were females, mean age was 14.7 months (SD = 7.1) and in 6% parents reported a disability.

**Table 1:** Characteristics of study participants and their child

Parameters	Total *N* = 769
Parental sex*, n* (%)	
Mother	677 (88.5)
Father	88 (11.5)
Age of respondent (y), mean (SD)	35.7 (4.3)
Education of survey respondent*, n* (%)	
Lower education	185 (24.2)
Higher education	580 (75.8)
Nationality*, n* (%)	
Swiss	530 (71)
Foreigner	217 (29)
Living in Partnership*, n* (%)	
Yes	755 (98.2)
No	14 (1.8)
Household net income (monthly)*, n* (%)	
Less than 4′500 CHF a month	34 (4.7)
Between 4′500 and 6′000CHF a month	99 (13.6)
Between 6′000 and 9′000CHF a month	240 (32.9)
More than 9′000 CHF a month	307 (42.1)
No indication/ don’t know	49 (6.7)
Sex of child*, n* (%)	
Female	377 (49.3)
Male	388 (50.7)
Age of child (m), mean (SD)	14.7 (7.1)
First child, *n* (%)	
Yes	377 (52.1)
No	346 (47.9)
Pregnancy week, mean (SD)	39.5 (2)
Birth weight of child (kg), mean (SD)	3.3 (0.5)
Child health status^a,^ *n* (%)	
Disability	42 (5.5)
No disability	722 (94.5)

Note: with exception of parental age with 143 missings (18%), missing values range from 0.5% up to a maximum of 7%

^a^defined as child with any of the following health problems: physical impairment of health (e.g. malformation), developmental delay, hearing or visual impairment, or congenital disability

### Use of digital media, print media, personal contacts and own experiences

The great majority (91%) of respondents reported using digital media when searching for information regarding their child’s health and development. Parents showed an equally high use of print media (92%) and all exchanged with at least one person of their formal or informal contacts when informing themselves on child’s health and development, irrespective of frequency of use. The most frequently reported digital media were by far search engines (55%) and webpages for parents (47%) (Table 2). Within our sample of digital media users, the median number of digital media used was 4 (IQR 3–5). Except for books, print media were not used very frequently in this population-sample. Digital media users reported slightly higher percentages of use of books when searching for health information about their child than non-digital users (31% vs. 25%). With regard to personal contacts, family members were were the most frequently used information source within this category as well as overall, for both digital media user and non-user. Compared to non-users, digital media users had higher proportions in informal personal contacts, e.g. for family (60% vs. 57%) and friends, neighbor, or acquaintances (50% vs. 44%). Exchange of information regarding child’s health and development with formal personal contacts, namely pediatrician, health professionals, or telephone consultation of a children’s emergency service or hospital was more frequent among non-users.

**Table 2:** Frequent use of information sources by parents, stratified by digital media users and non-users

Variables	Personal contacts				Variables	Print media				Variables	Digital media^a^	
	Users *N* = 700		Non-users *N* = 69			Users *N* = 700		Non-users *N* = 69			Users *N* = 700	
	N	%	N	%		N	%	N	%		N	%
Family	693	59.6	68	57.3	Books	694	31.1	68	25	Search engines	696	54.9
Friends, neighbor, acquaintances	689	49.9	66	43.9	Other print media	649	7.9	60	3.3	Webpages for parents	694	47
Pediatrician	690	31.6	68	41.2	Magazines	686	7.1	65	7.7	Official webpages of health services or health organizations	695	13.4
Other health professionals	680	18.7	63	23.8	Newspapers	686	3.1	63	0	Webpages of pediatricians or children’s hospitals	695	12.1
Telephone consultation of a children’s emergency service or hospital	682	4	63	6.4						Apps	691	7.4
Telephone consultation of health insurance	672	3.1	64	3.1						Social media	698	5.44

Note: group differences by digital users and non-users assessed by means of a chi2 non-significant in all cases

^a^Question only asked to digital media users

Half of the parents (51%) reported to ‘frequently’ or ‘very frequently’ refer to own past experiences, for example with an older child, with regard to child health and development of the youngest child. Parents with more than one child under 15 years reported to refer to previous experiences much more frequently (88%) compared to first-time parents (22%).

The proportion of individuals who frequently used digital media by parental sex, parental education, and first child are presented in Table 3. Lower education level was significantly associated with a higher use of social media (*p* = 0.040), and higher education with a higher use of websites targeted at parents (*p* = 0.019). Significant differences were also found between first time parents and parents with more than one child. The first group made higher use of webpages targeted at parents (56% vs. 37%, *p* < 0.000), apps (11% vs. 3%, p < 0.000), and webpages of pediatricians or children’s hospitals (16% vs. 8%, *p* = 0.005). A borderline significant result was found for search engines (58% vs 51%, *p* = 0.060) and official webpages of health services or health organizations (16% vs. 11%, *p* = 0.062). Mean age was significantly lower in frequent users of apps (M = 35 SD = 3.27) compared to non-frequent users (M = 35.8, SD = 4.19, *p* = 0.047). Similarly, official webpages of health services or health organizations, where frequently accessed by younger parents (M = 35.07 SD = 3.53 vs. M = 35.88 SD = 4.21, F (1,571) = 2.50), however, this result is of borderline significance (*p* = 0.055).

**Table 3:** Proportion of individuals frequently using digital media by sociodemographic characteristics

Digital media	Parental sex			Parental education			First child		
	Mother	Father	*P*	Lower education	Higher education	*P*	Yes	No	*P*
	%	%		%	%		%	%	
Social media	6	1.4	0.100	8.7	4.5	0.040	5.2	4.8	0.824
Webpages for parents	46.7	50.7	0.518	38.9	49.4	0.019	56.4	36.8	< 0.001
Apps	7.6	4.1	0.273	8.3	6.8	0.521	11.1	2.9	< 0.001
Search engines	54.8	55.4	0.926	60.4	53.4	0.119	58	50.7	0.060
Webpages of pediatricians or children’s hospitals	12.2	11	0.751	10.1	12.8	0.361	15.7	8.4	0.005
Official webpages of health services or health organizations	13.1	16.4	0.424	11.3	14	0.376	15.7	10.7	0.062

Note: *P*-values are derived from χ2-test for categorical data and ANOVA for numerical data. Parental sex category ‘others’ of survey respondents (*n* = 4) was excluded for this analysis. N’s of parental sex, parental education and first child ranged between 691 and 698

Few parents reported using social media, of any type, frequently for health information regarding their child’s health and development (5%). Amongst the social media listed, Facebook was the most often mentioned; 18% of parents using social media did so ‘frequently’ or ‘very frequently’ while 25% ‘sometimes’, and 22% ‘rarely’.

Our findings yield that parents do not use apps frequently when looking for information about the children’s health and development. Among the app-users 40% stated to use apps at least ‘rarely’ and 7% used apps ‘frequently’ or ‘very frequently’. The three most popular apps, irrespective of their frequency of use, were ‘*Oje, ich wachse!*’ (41%), an app on child growth, ‘*BabyCenter*’ (21%) and ‘*Swissmom*’ (18%), both apps about pregnancy and infancy.

The digital medium frequented the most, after search engines, are websites for mothers and fathers: almost all parents (96%) using digital media reported accessing websites to inform themselves on children’s health and development, and almost half (47%) consulted these sites frequently or very frequently. The most frequently selected websites were ‘*swissmom.ch’* (84%), ‘*babycenter.de’* (32%), ‘*wireltern.ch’* (32%), ‘*letsfamily.ch’* (29%), ‘*rund-ums-baby.de’* (23%), ‘*familienleben.ch’* (22%), *‘netmoms.de* (19%), and *‘urbia.de’* (18%).

The use of chats, posts or forums for child’s health and development was rather common, two-thirds (74%) stated to read entries written by other people. A small percentage of these parents (13%), actively participated in chats, posts or forums and either shared personal experiences, asking for opinions or responded to questions posted by other parents.

### Reasons for using or not using digital media

**Fig. 1:**
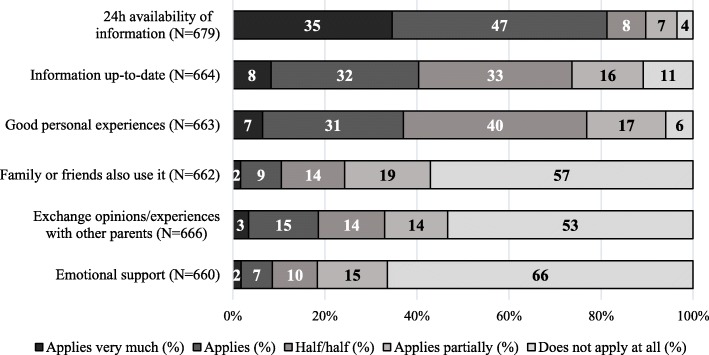
Reasons for using digital media

The most common reason for using digital media for information about general child’s health and development, was the 24/7 availability of information (82%, Fig. 1). The fact that information available online is up-to-date (40%) and good personal experiences with digital media (38%), was also mentioned as a reason for making use of digital resources. However, emotional support was not perceived as a relevant reason for going online.

The most common reasons for not using digital media were the difficulty of finding clear information on the Internet (46%) and not knowing which information is actually reliable (32%) (Table 4). A lack of Internet access was not a reason among the study population.

**Table 4:** Reasons for not using digital media for general health information

Variables^a^	Non-users *N* = 69
It is difficult to find clear information	46%
I do not know which information is reliable	32%
The information found is confusing / difficult to understand	12%
I do not know where / how to search	3%
No Internet access	3%
Other reasons mentioned by parents as free text:	
No need, because child is not sick or other good source of information available	17%
Untrustworthiness	10%
Uncertainty, fear, worry	7%
Personal preference	6%

^a^Respondents could select more than one option

### Internet as a good resource for improving health knowledge

More than two-third of digital media users agree that the Internet is a good resource for improving their knowledge on health related topics (76%, Table 5). Regarding information obtained from the Internet (Table 5), the great majority of respondents (91%) only sometimes believed the information to be correct. 64% reported to always try to assess the reliability of websites before using the information. About half (48%) stated to always understand the acquired health information and a similar proportion (46%) only sometimes understood the information received. The majority of digital media users (67%) would like receiving suggestions of reliable Internet sources from their pediatrician while among non-users less than half (41%) reported the wish for guidance.

**Table 5:** Trust in and assessment of online health information

Question	Answers given by digital media users (*N* = 700) *n* (%)					
	Completely agree	Rather agree	Rather disagree	Completely disagree	No indication/ don’t know	Not answered
*How much do you agree with the statement “the Internet is a good tool to help improve my knowledge of health-related topics”?*	88 (12.6)	442 (63.1)	110 (15.7)	21 (3)	7 (1)	32 (4.6)
	Always	Sometimes		Never	Not answered	
*How often do you believe that the information is correct?*	22 (3.1)	640 (91.4)		6 (0.9)	32 (4.6)	
*Do you try to find out if a site is reliable before accepting or using the information?*	446 (63.7)	159 (22.7)		60 (8.6)	35 (5)	
*How often are you sure you understand what you find on the Internet?*	337 (48.1)	318 (45.4)		4 (0.6)	41 (5.9)	
	Yes	No		Don’t know	Not answered	
*Would you like your pediatrician to give you trustworthy Internet resources?*	467 (66.7)	124 (17.7)		80 (11.4)	29 (4.2)	

Note: n’s not summing up to 700 due to missing data

The explorative regression analyses yielded significant results for the factors parental sex, internet use for health-related information in the last 12 months and frequency score, adjusting simultaneously for all independent variables in the regression model (Table 6).

**Table 6:** Explorative analyses of factors associated with parental perception of the Internet being a good resource

Independent variables	OR	95% CI	*P*
Parental characteristics			
Education [ref. lower education]	1.29	0.87–1.92	0.207
Father [ref. mother]	2.09	1.15–3.78	0.015
First child [ref. no]	0.78	0.55–1.10	0.154
Child’s characteristics			
Disability [ref. no]	1.2	0.62–2.32	0.587
Age	1.01	0.99–1.04	0.274
Online behavior of parents			
Used Internet for health-related info last 12 months [ref. no]	2.15	1.28–3.64	0.004
Frequency score of digital media use	1.15	1.09–1.21	< 0.001
N	614		
R^2^	0.048		< 0.001

Note: the dependent variable distinguishes between (1) completely disagree, (2) rather disagree, (3) rather agree and (4) completely agree, an increased OR indicates a higher agreement level

Among the parental and child characteristics, only parental sex yielded significant effect estimates. Fathers had higher odds of perceiving the Internet as a good resource compared to mothers (OR = 1.80, 95% CI: 1.03–3.16). The sensitivity analysis with the variable general use of digital media (yes/no) and the one without the frequency score showed similar results (see Additional file 2). Significant effects sizes were found for the binary variable Internet use in the last 12 months. Parents who had resorted to the Internet when searching health-related information during the last year were more likely to report a higher agreement level with the statement “the Internet is a good tool to help improve my knowledge of health-related topics” (OR = 2.89, 95% CI: 1.86–4.50). Individuals with a higher frequency of use of digital media when searching for child’s health and development information were 1.15 times more likely to perceive the Internet as a good resource for improving their health knowledge (OR = 1.15, 95% CI: 1.09–1.21).

## Discussion

This first-time survey on the digital health information seeking behavior in Swiss parents yields a high proportion of parents using the Internet frequently to inform themselves on children’s health and development. Search engines and webpages for parents were by far the most frequently used digital media. Generally, the internet is perceived as a good resource, however, a large percentage of parents is skeptical about the correctness and their own understanding of the health information received.

Differences in use between digital media, personal contacts, and print media among our study population were small and all three sources were frequently used. Family members, followed by search engines, friends, or acquaintances, webpages for parents, pediatrician, and books were among the most frequented sources for general pediatric information. These results support findings from other countries that digital media are complementary rather than substitute of traditional sources of health information [25–27]. This interpretation is further sustained by the results of a multinational study in pregnant women which found that expectant mothers use multiple sources when searching for information including health professionals, different print media and the Web [28].

Previous studies on the adoption of digital media for seeking information on children’s health and development to parents are mostly from English-speaking countries. Similar to our results, these studies identify search engines as the most popular way of finding pediatric health information [10, 11, 27]. However, only about half of the Swiss parents access search engines for this purpose, compared to the UK where 75% of parents [29] and Norway where 96% of mothers [27] use Google for their searches. Search engines, however, are a random entry door to further sites. Eysenbach et al. [30] observed that people often only explore the first webpages shown by search engines and afterwards return to these webpages [10].

Parenting apps and social media were used infrequently by our study population; only 7% of parents used apps and 5% social media, far lower compared to other countries. In an Australian study mothers’ app use was particularly common; 49% of mothers resorted to parenting apps, of which 19% used the apps daily and 15% a few times a week [7]. Pregnant mothers in the same study used pregnancy apps even more often (73%). Compared to English-speaking countries, the choice of apps developed for Swiss parents is scarce, thus the lower frequency of use may be due to the lack of offers and choice, and less to a lack of interest.

Earlier studies underline that social media are used to create a social network, establishing connections with other parents and exchanging or sharing information [31–33]. Thus a main motive for its use is emotional support [34]. Parents in our survey did not consider emotional support a relevant reason, which corresponds to the infrequent use of social media in our survey. The low use may also relate to changes in popularity of social media as well as digital applications. Swiss market analysists indicate increasing popularity and frequency of use of short messaging social media such as Whatsapp or Instagram in younger adults < 35 as compared to older adults [35]. These short message social media are possibly less suitable for seeking health information than other social media.

A number of studies have explored the digital divide and associations between socio-demographic characteristic and online health information seeking. Overall, they document a less frequent use of online health information by individuals with lower educational level or socio-economic status than those with higher education or socio-economic status [36–38]. A digital divide may also occur due to lower digital literacy in lower educated populations [39], reducing the chances that relevant information might be found and processed in a correct way. Our data indicate that individuals with a higher educational level reported more frequent use of webpages targeting parents [40], while social media was used more frequently by individuals with lower education, especially by participants with compulsory schooling only. It is thus relevant to provide high-quality information on all digital media to ensure access for all parents, irrespective of their preference and to enhance the digital health literacy of parents.

First-time parenthood was associated with significant higher use of all digital media in our study. This indicates a particular parental need of information and support in the postnatal period, as has been suggested by other authors [6, 41]. With regard to parity, however, literature is inconsistent. Bernhardt et al. [10] report higher use of the Web in the U.S by first-time mothers with a child presenting symptoms compared to mothers with more than one child, while no differences by parity was found in a study from Norway [27].

The Internet provides the possibility to access a wide range of information rapidly, easily, and privately [26]. Our results, in fact, indicate that the 24/7 availability is the most important reason for which Swiss-German parents use digital media. A focus group with Australian mothers also found that parents appreciate ready and immediate information [42].

Assessing the accuracy of online information is not as simple, and parents’ evaluation of the trustworthiness is often suboptimal [30]. Much of the online information is complex and requires a higher literacy level [43], and many parents have difficulty understanding common pediatric health information [44, 45]. A higher educational level has been associated with a higher health literacy [46], and higher Internet skills [47]. Most participating parents were rather skeptical about the health information they find on the Internet. It is reassuring that almost two-thirds of the study population stated to always check the reliability of the website, however, more than a third did not or only sometimes. Compared to an Australian study by Wainstein et al. from 2006 [9], participants of this Swiss study were much more doubtful about the trustworthiness of the information: only 3% vs. 23%; always believe the information and 64% vs. 45% always checked the reliability of a source. Half of the Swiss parents always understood the health information found online as compared to a third in Australia. Although still lower than in Switzerland, a more recent study from Australia [48] found a higher percentage of parents expressing concerns about the reliability of online information than by Wainstein et al. [9], indicating that increasing experience of societies with the Internet may change their evaluation of risk and benefits. Surprisingly, this sceptic attitude, reported by our study population, does not lead to a higher access of trustworthy websites, such as websites by children hospitals, pediatricians, or health services. Possibly, these sites are harder to find via search engines. In fact, two-thirds expressed the wish, that their pediatrician would recommend trustworthy resources. Swiss parents, however, seem to realize their need of guidance less than in the UK, for example, where 88% of parents felt that doctors should suggest reliable online sources compared to 66% in Switzerland [49]. Addressing the wish for guidance by practitioners and public health experts could greatly increase the overall digital health literacy among parents and public health potential of digital media. Prior to further promotion of digital media we recommend to increase professional eHealth competencies, as professionals will be increasingly encountered with questions and recommendations, and quality of digital information sites. Health professionals may also consider alternative communication channels. As illustrated by an Italian study, only a minority of general practitioners offers patients the opportunity to communicate via instant messaging apps [50].

Although parents were aware that digitally obtained information might not always be correct and their understanding might be limited, the Internet was generally perceived as a good resource for improving personal health knowledge. Parental sex was the only socio-demographic variable significantly associated with this perception; fathers were more likely to perceive the Internet as a good resource compared to mothers. Despite the continued presence of gender roles in child care, fathers are increasingly involved in taking care of their children [51]. However, Swiss fathers are not entitled to a paternity leave by law, and most continue or return to full-time work immediately after the birth of their child [52], which may, on average, restrict their personal contact to the child’s health professionals. Fathers are rarely addressed as a target group in research on digital media behavior in relation to children’s health and development. The few studies that did focused mostly on social networks like Facebook or forums [31]. A Swedish study by Fletcher et al. [53] also concludes that the Internet seems to be a suitable medium to reach fathers and providing them with useful information and support concerning their parental role. In fact, promotion of digital media to improve health literacy may be especially successful in fathers. Further, our results indicate that familiarization and previous digital experience in seeking health information is a main factor for appreciating the Internet as a good resource. The more frequent parents used the Internet for themselves, the more they agree it to be a good resource to improve their health knowledge.

There are some study limitations that may affect the generalization of results. The data were self-reported, which always carries the risk of reporting bias, and social desirability may play a role. However, we see no reason why parents would misreport on their use or competencies with respect to digital media. The study sample was drawn randomly from birth registries, but self-selection into the study generated an overrepresentation of highly educated parents. Three quarters of parents in our sample had a tertiary education, attained by slightly more than a third in the general Swiss population, aged 25–44, and only 3% had a compulsory education, 9 years of schooling, as compared to 10% in the general population [54]. Additionally, parents who were already interested in the topic and have a preference of using digital media for children’s health may have participated more readily.

Age of mothers at first birth in our sample was slightly higher than the general population (34.6 vs. 30.8) [55], which corresponds to the higher education level of the participants. With respect to household income the sample seems quite representative; the mean household income in the Canton of Zürich was 8677 CHF and the median category in our sample was between 6000 CHF and 9000 CHF [56]. The study was performed in the Swiss-German part of the country. Although comparisons with international studies yield similar conclusions, we cannot rule out that digital health seeking behavior might be different in other regions of Switzerland or in parents of different cultural background. It is noteworthy, that even though the study was in German, thus potentially excluding parents less fluent in the language, we reached a considerable percentage of parents with a migrant background (29%), similar to the national percentage [57]. In fact, we consider the participation of 30% in an epidemiological study that provides no incentive and addresses parents of small children more than acceptable. The questionnaire covered a large scope of questions around the parental use and perception of digital media and provides relevant results for public health practice and future research. We suggest further research in less-accessible and possibly more vulnerable populations, such as less educated and populations recently migrated to Switzerland.

## Conclusion

The Internet has become a relevant source of information for parents for general information on children’s health and development. Nevertheless, parents continue to use traditional resources, print media, and personal contacts. While personal social networks are frequently addressed in case of health questions, overall digital social networks play a minor role.

Although digital media are used frequently, parents report insecurity with respect to reliability and their understanding of the information, which could have important implications on children’s health and development. Health professionals should learn to support parents in the digital jungle of information. While fathers should be considered a specific target group for digital content, increased access to, knowledge about and promotion of high quality digital information sources will help satisfy both parents’ increased need of information during infancy and beyond, increase parental digital health literacy and, ultimately, improve children’s health.

## Additional files

Additional file 1: Questionnaire. (XLSX 21 kb)

Additional file 2: **Table S1.** Explorative ordered regression analyses. (XLSX 29 kb)

## Abbreviation

CHF: Swiss francs
